# The relationship between microbial population ATP and quantitative PCR bioburdens in diesel fuel microcosms

**DOI:** 10.1099/acmi.0.000695.v4

**Published:** 2024-07-04

**Authors:** Frederick J. Passman, Jordan Schmidt, Danika Nicoletti

**Affiliations:** 1 Biodeterioration Control Associates, Inc., PO Box 3659, Princeton, NJ 08540-3659, USA; 2 LuminUltra Technologies Ltd, 819 Royal Road, Building B, Fredericton, NB E3G 6M1, Canada

**Keywords:** ATP, bioburden, diesel, fuel, qPCR

## Abstract

Historically, fuel microbiology studies have relied on culture data. Potentially relevant but unculturable bacteria were not detected. Although ATP can quantify total microbial bioburdens in fuels, it cannot differentiate among the taxa present. Quantitative PCR (qPCR) testing promises to fill this gap by quantifying targeted amplicon sequences thereby detecting both culturable and non-culturable taxa and quantifying specifically targeted taxa. In this study, fluid samples drawn from the fuel, interface and water phases of fuel over water microcosms were tested for cellular ATP concentration ([cATP]) and qPCR bioburdens. Additionally, surface swab samples from steel corrosion coupon surfaces exposed to each of these three phases were collected and tested for total ATP concentration ([tATP]) and qPCR bioburdens. Statistical relationships between ATP and qPCR bioburdens were examined. Correlation coefficients between the two variables were matrix dependent and ranged from negligible (|*r*|=0.2) to strong (|*r*|=0.7). When results were categorized into negligible, moderate and heavy bioburdens, parameter agreement was again matrix dependent. Percentage agreement between [ATP] and qPCR gene copies ranged from 11 % to 89 % – with qPCR-bioburden ratings typically being greater than ATP-bioburden ratings.

## Data Summary

 The authors confirm that all supporting data have been provided within the article or through supplementary data files. Five supplementary tables are available with the online version of this article. Table S1 (available in the online version of this article) provides the data from which qPCR repeatability precision was calculated. Tables S2–S5 contain data from which summary data in the paper's figures and tables were derived.

## Introduction

Fuel biodeterioration was first reported more than 125 years ago [1]. Subsequently, a substantial body of fuel and fuel system biodeterioration literature has accumulated [2]. Typical fuel biodeterioration phenomena include metabolic consumption of C_4_ to C_7_ hydrocarbons, β-oxidation of higher molecular weight hydrocarbons and metabolic degradation of fuel additives. Metabolically active microbiological consortia in fuel systems produce low molecular weight (C_1_ to C_6_) organic acids and diverse biosurfactants. The introduction of biobased fatty acid methyl ester diesel fuel provided an additional nutrient source for micro-organisms growing in diesel fuels [3,4]. Consequently, fuels can become corrosive due to increased water content and acidity. Fuel oxidative stability decreases, and polymerized oxidation byproducts become a major component of particulate loads that can plug fuel filters. Except for long-term (>1 year) storage facilities, fuel biodeterioration has been demonstrated primarily in laboratory microcosms. However, microbiologically influenced corrosion of fuel infrastructure components, sensor fouling and premature filter plugging have become increasingly common phenomena [5,6].

Fuel and fuel system biodeterioration share common symptoms with abiotic processes [7]. Moreover, heavy bioburdens have been recovered from fuel systems with no apparent evidence of biodeterioration. Conversely, fuel systems with substantial gross evidence of biodeterioration [2] have provided below detection limits or negligible yields (i.e. greater than limit of detection but less than the control limit for an attribute designation of moderate contamination). One explanation between the apparently weak connection between observed bioburdens and biodeterioration is heterogeneity of microbial contamination distribution [8]. Microbes are only metabolically active where free water is available. Free water zones in fuel systems are often inaccessible for sampling. Moreover, micro-organisms involved in fuel biodeterioration are commonly present within surface-attached (sessile) communities in the form of biofilms. Biofilms accumulate on tank wall (shell) surfaces, at the fuel–water interface, and in bottom sediments. Thus, the apparent absence of aggressive microbiological communities in fuel system samples can be an artefact of samples coming from locations where microbial contamination is absent. Another explanation relates to population metagenomics, transcriptomics, metabolomics and proteomics [9]. A given bioburden can comprise aggressive, moderate or non-biodeteriogenic microbes, or any combination of these. Genomic testing can detect whether potentially relevant, deteriogenic genes are present. Transcriptomic, metabolomic and proteomic tests help to assess whether biodeteriogenic genes are active [10].

Prior to 2010, the primary parameter used to quantify microbial contamination in fuels and fuel systems was culture testing [2]. More recently, non-culture-based standard test methods have been developed [11,13]. Additionally, protocols for quantitative PCR (qPCR) [14] and next-generation sequencing genomic profiling [15] have been developed. As more field studies using non-culture methods are performed, the relative contributions of sampling challenges, and population characteristics to microbiology test results are likely to become disambiguated.

In industrial fuel storage systems, fuel condition monitoring includes testing for microbiological contamination [16,17]. As noted by Passman [2], historically, the most common microbiological parameter tested has been culturability [18]. Any given culture test will only detect microbes that will proliferate under the conditions of the protocol. Nutrient composition, atmosphere and temperature select for a subset of the total microbiome. Consequently, reliance on culturability is likely to underestimate microbial loads in fuels and fuel-associated waters [19].

The time required to complete an ASTM D7687 test is <10 min. However, qPCR testing requires approximately 2 h to complete. Both protocols can be performed near the sample collection site [20]. The minimal delay between sample collection and data availability allows operators to initiate remedial treatments in a timelier manner – often on the same day samples are collected. Demonstrating test result agreement among non-culture methods is an essential step in new method adoption. Previous studies have demonstrated strong agreement between ATP and culture data [20,21], and between ATP and immunoassay data [13].

Recently, Passman *et al*. [22] reported on the relationship between cellular ATP concentrations ([cATP]) and adenylate energy charge (AEC) in fuel over water microcosms. This paper reports the relationship between ATP and qPCR-based planktonic and sessile bioburden quantification in diesel fuel, bottom-water and inverted emulsion layers in microcosms aged for 18 months. This is the first published comparison between ASTM Test Method D7687 and qPCR data from fuels and fuel-associated water.

## Methods

### Microcosms

The microcosm configuration and test array have been detailed previously [15,22]. Briefly, 67, 2 litre, wide-mouthed glass jars were filled with 1300 ml diesel fuel over 250 ml Bushnell-Hass salts medium [23]. Half of the microcosms were inoculated with a challenge population enriched from microbiologically contaminated underground storage tanks from a retail site. All microcosms were stored in the dark, in fume hoods, at laboratory room temperature (20±2 °C).

### Bioburden testing

Bottom-water and fuel phase sample cATP (pg m^l−1^) were determined by the ASTM D7687 Standard Test Method for Measurement of Cellular Adenosine Triphosphate in Fuel and Fuel-associated Water With Sample Concentration by Filtration [11]. This test method was developed to eliminate interference that previously prevented ATP from being a reliable parameter for testing bioburdens in fuel. Briefly, a fuel or fuel-associated water specimen is filtered to trap contaminant microbes onto an in-line glass fibre filter. The filter retentate is then washed with a proprietary solution to remove organic and inorganic molecules known to interfere with luminometry. After washing and air drying, the retained cells are lysed and the extracted ATP is flushed into a buffer solution. Finally, 100 µl of ATP extract is mixed with 100 µl of a Luciferin-Luciferase reagent and the resulting bioluminescence reaction is quantified as relative light units (RLU) by luminometry. Luminometric results from test specimens are compared against RLU from a 1 ng ml^−1^ ATP reference standard and converted to [cATP] in pg ml^−1^. In this study, ASTM D7687 was modified in that 5 ml fuel and 1 ml aqueous specimens were tested instead of the 20 ml fuel and 5 ml water specimens prescribed in D7687. Surface (biofilm) was tested using 1×1 cm swab samples. After sample collection, total ATP (tATP) concentration ([tATP], as pg cm^−2^) was determined by ASTM Test Method E2694 using a deposit and surface analysis method described previously [24,25]. The [cATP] and [tATP] values were transformed to log_10_ pg ml^−1^ and log_10_ pg cm^−2^, respectively.

A commercially available qPCR test system (GeneCount, LuminUltra Technologies) was used to quantify total prokaryote (TP) and total fungal (TF) gene counts – reported as gene copies (GC) ml^−1^ and GC cm^−2^ for liquid and surface samples, respectively. For comparison with ATP-bioburden data, qPCR results were log_10_ transformed and given categorical scores.

### Statistical analysis

The coefficient of variation (CV), standard deviations (*s*_r_) and the Pearson two-sided correlation coefficient (*r*) were computed using the Microsoft Excel Version 16.0 (Microsoft) Excel Analysis ToolPak Add-in. The linear regression (*r*^2^) values for the scatter plots were calculated using the lm function of the R package ggplot2 (R Version 4.3.2), and the 95 % confidence intervals of the trend lines were generated using the stat_smooth function of the same R package.

## Results and discussion

### Test method precision

Repeatability precision for ATP testing has been reported previously [11,24]. A subset of samples was tested for TP and TF repeatability precision per ASTM Practice E1601 [26]. The data are provided in Table S1. Repeatability standard deviations (*s*_r_) for log_10_ TP GC ml^−1^ and log_10_ TF GC ml^−1^ were 0.24 and (CV=4.8 %) and 0.11 (CV=2.8 %), respectively.

### Aqueous phase

The limits of detection (LOD) for [cATP] and qPCR were 0.5 pg ml^−1^ (−0.3log_10_ pg ml^−1^) and 20 GC ml^−1^ (1.3log_10_ GC ml^−1^). Recognizing that the ATP test does not differentiate between bacterial and fungal cATP, ATP-bioburdens were compared with the sum of total prokaryote and total fungal qPCR-bioburdens. Specimens for which qPCR values were below the detection limit were included in this analysis as this result does not negate the presence of the gene target. qPCR values below the detection limit could reflect reaction inhibition, or insufficient template DNA. One common cause of insufficient template DNA recovery is inadequate cell lysis [27].

**Fig. 1.**
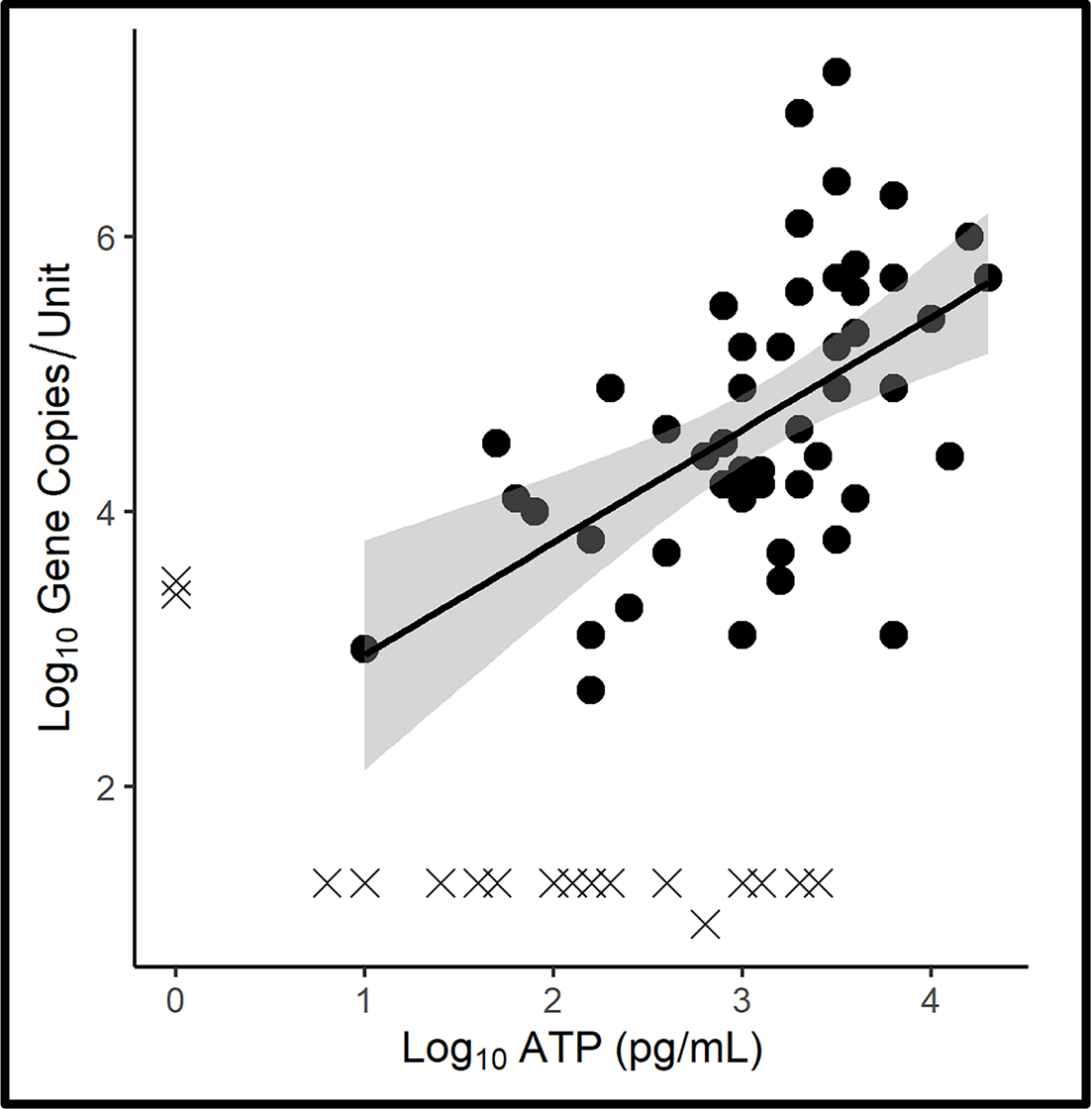
Microcosm aqueous-phase ATP-bioburden versus qPCR-bioburden scatter plot. Grey shading along the trend line indicates the 95 % confidence interval. Datapoints denoted with ‘X’ are at or below the limit of detection (LOD) for qPCR or ATP. These values were excluded from the trendline and 95 % confidence interval as values below the LOD do not follow the same probability of repeated detection as datapoints within the range of detection.

Raw and categorized ATP and qPCR bioburden data are provided in Table S2. The summary statistics shown in Table 1 demonstrate that inoculation with a challenge population did not impact either ATP or qPCR recoveries in aged microcosms. Fig. 1 shows a scatter plot of the planktonic, aqueous phase ATP and qPCR data with a positive trend line [*y*=0.82*x*+2.14, *r*^2^ : 0.3; where *y* is log_10_ GC ml^−1^ and *x* is log_10_ [cATP] (pg ml^−1^)]. The Pearson, two-sided correlation coefficient (*r*) between log_10_ [cATP] and log_10_ GC ml^−1^ was 0.57, where *r*_crit [df =63; α=0.05]_=0.21. Among the 67 microcosms, 36 were inoculated with an uncharacterized microbial enrichment developed from high-bioburden ([cATP]>4 log_10_ pg ml^−1^) UST bottom-water samples and 32 were uninoculated. It was reported previously that within 12 weeks, there was no statistical difference between ATP-bioburdens in inoculated versus uninoculated microcosms [15].

**Table 1.** Inoculation impact on log_10_ cATP and qPCR bioburdens in aqueous-phase samples

Parameter	Uninoculated		Inoculated			
	Aerage	*s*	Average	*s*	*F*_obs_*	*P*-value
[cATP]	3	1.3	3.0	0.66	2.63	0.11
TP qPCR	4	1.9	4	1.8	0.049	0.82
TF qPCR	2	1.6	2	1.2	1.16	0.29
TP+TF qPCR	4	1.7	4	1.7	1.49	0.23

Note a – *F_crit [1,66; α=0.05]_=3.99.

sstandard deviation

Traditionally, microbiological condition monitoring test data from fuels and fuel-associated waters have been assigned to classification categories (*negligible*, *moderate* and *heavy* contamination) with associated categorical scores (1 – negligible, 3 – moderate and 5 – heavy) [28]. Assignment to classification categories counters end-user tendencies to overinterpret data (i.e. inappropriately inferring significant differences among results that are within a given test method’s 95 % confidence reproducibility limits). Categorical scores facilitate comparisons among microbiological test methods [13].

Classification categories are provided in Table 2. For ATP-bioburdens, categories are based on previously established culture-based ranges [28] and unpublished [cATP] versus c.f.u. ml^−1^ calibration curves. Consequently, the same categorical threshold values were used for both cATP and qPCR bioburden measurements.

**Table 2.** Categorical (attribute) scores for aqueous phase cATP and qPCR bioburdens (values are log_10_ pg ml^−1^ and log_10_ GC ml^−1^ for liquid samples and log_10_ pg cm^−2^ versus log_10_ GC cm^−2^ for surface samples)

	Categorical designation (attribute score)		
Sample type	Negligible(1)	Moderate(3)	Heavy(5)
Aqueous	<2.0	2.0 to <4.0	≥4.0
Fuel	<1.0	1.0 to <2.0	≥2.0
Inverted emulsion	<3.0	3.0 to <5.0	≥5.0
Coupon surface	<3.0	3.0 to <5.0	≥5.0

Table 3 summarizes categorical score agreement, by test method, for all 66 microcosms and by whether microcosms were inoculated intentionally. Overall, the ATP and qPCR bioburden attribute scores agreed for 36 (55 %) of the samples. For eight (12 %) samples the ATP-bioburden score was greater than the qPCR-bioburden score and for 21 (31 %) samples the qPCR-bioburden score was greater than the ATP-bioburden score. Because qPCR is a molecular-based method that targets specific genes the method does not distinguish between active and inactive (moribund or dormant) cells. This is indicated in the attribute score comparison where qPCR was more likely to show a higher attribute score than ATP for the same sample. The qPCR-based categorical score was greater than the ATP-based score in 13 of 31 uninoculated (42 %) microcosms. Categorical score agreement was substantially greater among inoculated microcosms. Additionally, in inoculated microcosms the qPCR-based score was greater than the ATP-based score considerably less frequently.

**Table 3.** Parameter attribute score agreement – aqueous-phase ATP-bioburden and qPCR-bioburden

Data set	ATP=qPCR		ATP>qPCR		ATP<qPCR	
	No.	%	No.	%	No.	%
All microcosms	40	60	19	28	8	12
Uninoculated	20	61	7	21	6	18
Inoculated	20	59	12	35	2	6

ATP and qPCR results were compared for 65 microcosms, including 34 inoculated and 31 uninoculated microcosms.

The [cATP] in metabolically active micro-organisms can range from 0.5 to 6.5 fg per cell but typically averages 1.0 fg per cell [29]. The [cATP] in dormant micro-organisms is undetectable. Portnoï *et al*. [30] estimated that the DNA concentration per bacterial cell ranges from 1.5 to 4 fg. This estimate corroborates the *y*=0.82*x*+2.14 regression equation for the ATP-bioburden versus qPCR-bioburden scatter plot trend line in Fig. 1.

Table 4 summarizes one-way ANOVA of the ATP and qPCR data for five sample types: planktonic aqueous, planktonic fuel, planktonic interface, corrosion coupon surface aqueous and corrosion coupon surface interface. All ATP and qPCR corrosion coupon surface sample results from coupon areas exposed to fuel were below detection limits.

**Table 4.** ANOVA summary – relationship between [cATP] and qPCR bioburdens in fuel over water microcosm samples

Sample type	**No. of samples tested**	d.f.	*F*_obs_	*P*	*F*_crit_ (α=0.05)
Aqueous	68	134	22	6.0E-05	3.91
Fuel	9	16	3.1	9.6E-02	4.49
Interface	21	40	8.6	5.6E-03	4.08
Coupon–aqueous	9	16	25	1.4E-04	4.49
Coupon–interface	9	16	11	4.4E-03	4.49

The ANOVA statistics indicated that although ATP and qPCR-bioburdens correlated significantly and their respective categorical scores agreed for 55 % of the samples tested, qPCR-bioburdens (mean=4±1.7 log_10_ GC ml^−1^) were generally greater than ATP-bioburdens (mean=3±1.0 log_10_ pg ml^−1^). Previously, Martin-Sanchez *et al.* [31] compared culture and qPCR data from bottom-water samples collected from six forecourt underground storage tanks. Bacterial and fungal culture recoveries ranged from 3.5 to 6.0 log_10_ c.f.u. ml^−1^ and from 1.9 to 3.8 log_10_ c.f.u. ml^−1^, respectively. Although ASTM D8412 [14] prescribes reporting qPCR results in GC ml^−1^, Martin-Sanchez *et al.* reported their qPCR results as [DNA] in ng ml^−1^. Consequently, the forecourt bottom-sample results cannot be compared with the qPCR results from microcosms as reported in this paper. Martin-Sanchez *et al.* reported [DNA]_bacteria_ ranging from 99 to 246 ng ml^−1^ and [DNA]_fungi_ ranging from 0.1 to 29 ng ml^−1^.

### Fuel phase

**Fig. 2.**
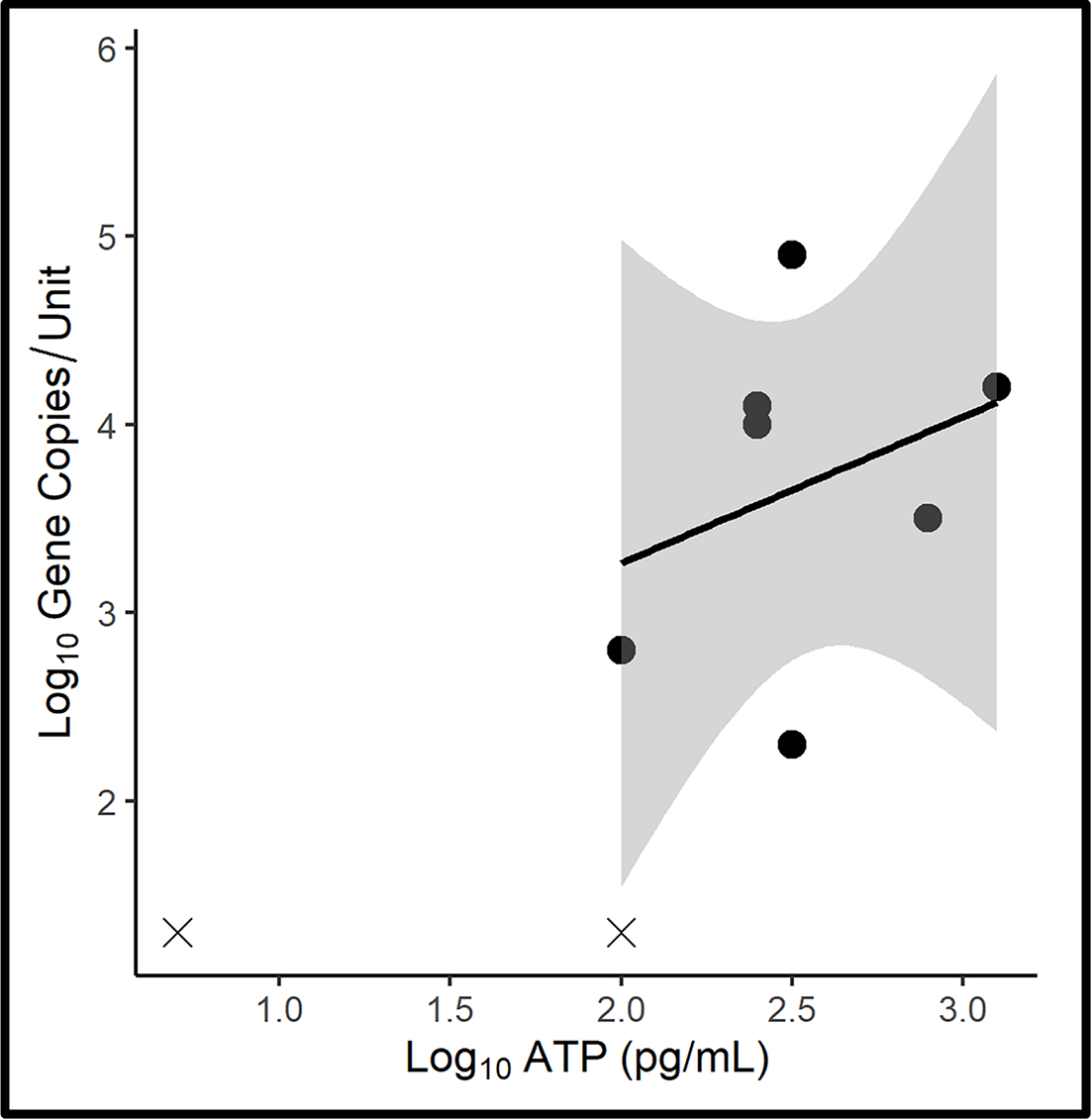
Microcosm fuel-phase ATP-bioburden versus qPCR-bioburden scatter plot. Grey shading along the trend line indicates the 95 % confidence interval. Datapoints denoted with ‘X’ are at or below the limit of detection (LOD) for qPCR, or ATP. These values were excluded from the trendline and 95 % confidence interval as values below the LOD do not follow the same probability of repeated detection as datapoints within the range of detection.

It was anticipated that fuel phase bioburdens would be negligible. Consequently, specimens for fuel-phase ATP and qPCR-bioburden testing were collected from only nine microcosms. Bioburdens were negligible in only one sample. The small sample size (nine samples) renders any statistical analysis tenuous. The raw data are provided in Table S3 and a scatter plot of the results and positive trendline [*y*=0.78*x*+1.71, *r*^2^: 0.1; where *y* is log_10_ GC ml^−1^ and *x* is log_10_ [cATP] (pg ml^−1^)] are shown in Fig. 2. The categorical scores for the two parameters for fuel samples from four of the nine microcosms (44 %) were compared. The qPCR-based scores were greater than ATP-based scores for four of the remaining microcosms (44 %). It is generally believed that micro-organisms suspended in fuel are dormant unless there is free water present. Haze ratings [15] and AEC data [13] were available for four of the microcosms. In the two microcosms with haze rating=2 [32] both ATP and qPCR bioburdens were approximately 2 log_10_ pg or GC ml^−1^ and AEC=0.42±0.05 – indicative of a highly stressed population. In contrast, in the two microcosms with haze rating=5, ATP-bioburdens were 3.01±0.099 log_10_ pg ml^−1^ and qPCR-bioburdens were 3.9±0.49 log_10_ GC ml^−1^. Although these observations were limited, they support the hypothesis that microbial activity in fuels is proportional to the availability of free water as indicated by fuel haze.

As per Table 4, qPCR (mean=3±1.3 log_10_ GC ml^−1^) and ATP-based (mean=2.3±0.69 log_10_ pg ml^−1^) bioburdens were not significantly different.

### Interface

**Fig. 3.**
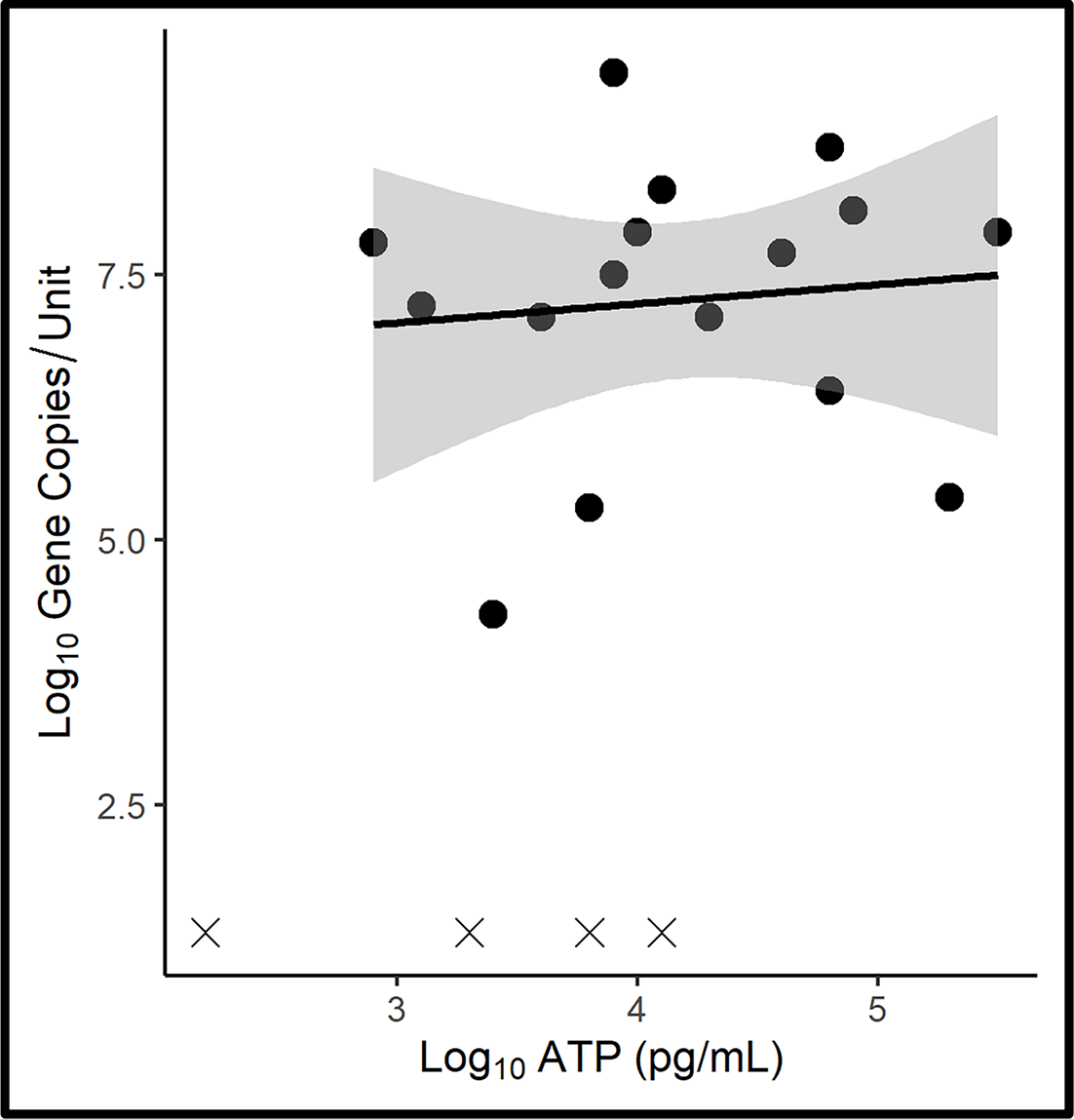
Microcosm interface ATP-bioburden versus qPCR-bioburden scatter plot. Grey shading along the trend line indicates the 95 % confidence interval. The datapoints denoted with ‘X’ are at or below the limit of detection (LOD) for qPCR or ATP. These values were excluded from the trendline and 95 % confidence interval as values below the LOD do not follow the same probability of repeated detection as datapoints within the range of detection.

The raw interface data are provided in Table S4 and a scatter plot of the results is presented in Fig. 3 with Fig. 3 positive trend line [*y*=0.18*x*+6.5, *r*^2^: 0.1; where *y* is log_10_ GC ml^−1^ and *x* is log_10_ [cATP] (pg ml^−1^)]. The Pearson, two-sided correlation coefficient (*r*) between log_10_ [cATP] and log_10_ GC ml^−1^ was 0.52, where *r*_crit [df=19; α=0.05]_=0.54, indicating that the relationship between the two parameters was not significant. Overall, qPCR-bioburdens (mean=6±2.8 log_10_ GC ml^−1^) were two orders of magnitude greater than ATP-bioburdens (mean=3.9±0.90 log_10_ pg ml^−1^). The qPCR-based categorical scores were greater than the ATP-based scores for 62 % (13 of 21) of samples. These results suggest that a substantial fraction of the interface population is dormant or dead (metabolically inactive). However, Passman *et al.* [13] found that the interface AEC was 0.76±0.1 – significantly greater (*P*_0.95_=7.5×10^−7^) than the aqueous phase AEC (0.57±0.3).

**Fig. 4.**
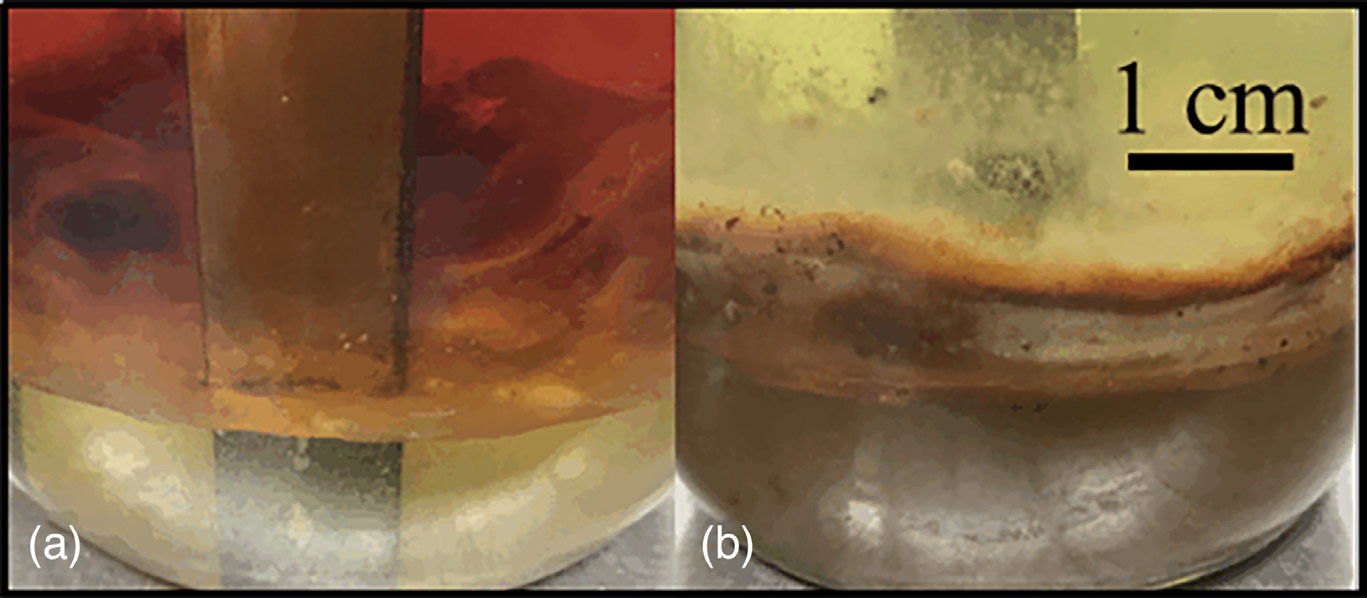
Fuel–water interface in diesel over Bushnell-Haas medium microcosms: (a) the interface zone is <1 mm thick; (**b**) the interface zone is >1 cm thick.

The interface between fuel and bottom-water in microbially contaminated fuel systems can vary substantially (Fig. 4). In some systems, microbial growth forms a mono-dimensional layer (Fig. 4) and in others it can create a >1 cm thick pellicle consisting of extracellular polymeric substance and inverted emulsion micelles. The ratio of metabolically active to inactive cells is known to vary substantially among biofilm communities [33]. Consequently, the relationship among microbiological parameters in the fuel–water interface zone is expected to be more variable than in either the fuel or aqueous phases.

### Corrosion coupon surfaces

**Fig. 5.**
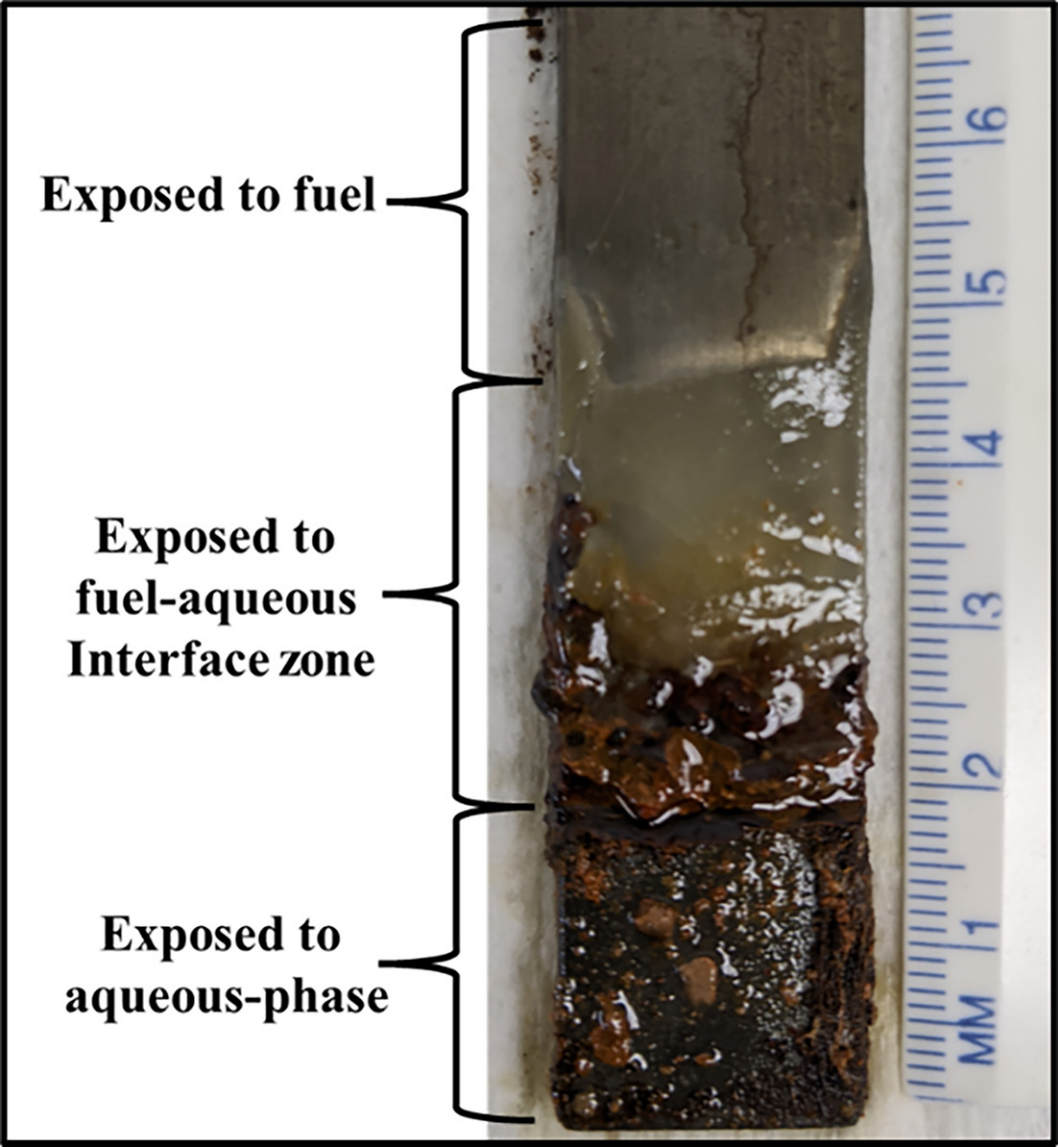
Biofilm accumulation on mild steel corrosion coupon immersed in fuel over Bushnell-Haas medium microcosm. Heaviest accumulation was in the interface zone.

**Fig. 6.**
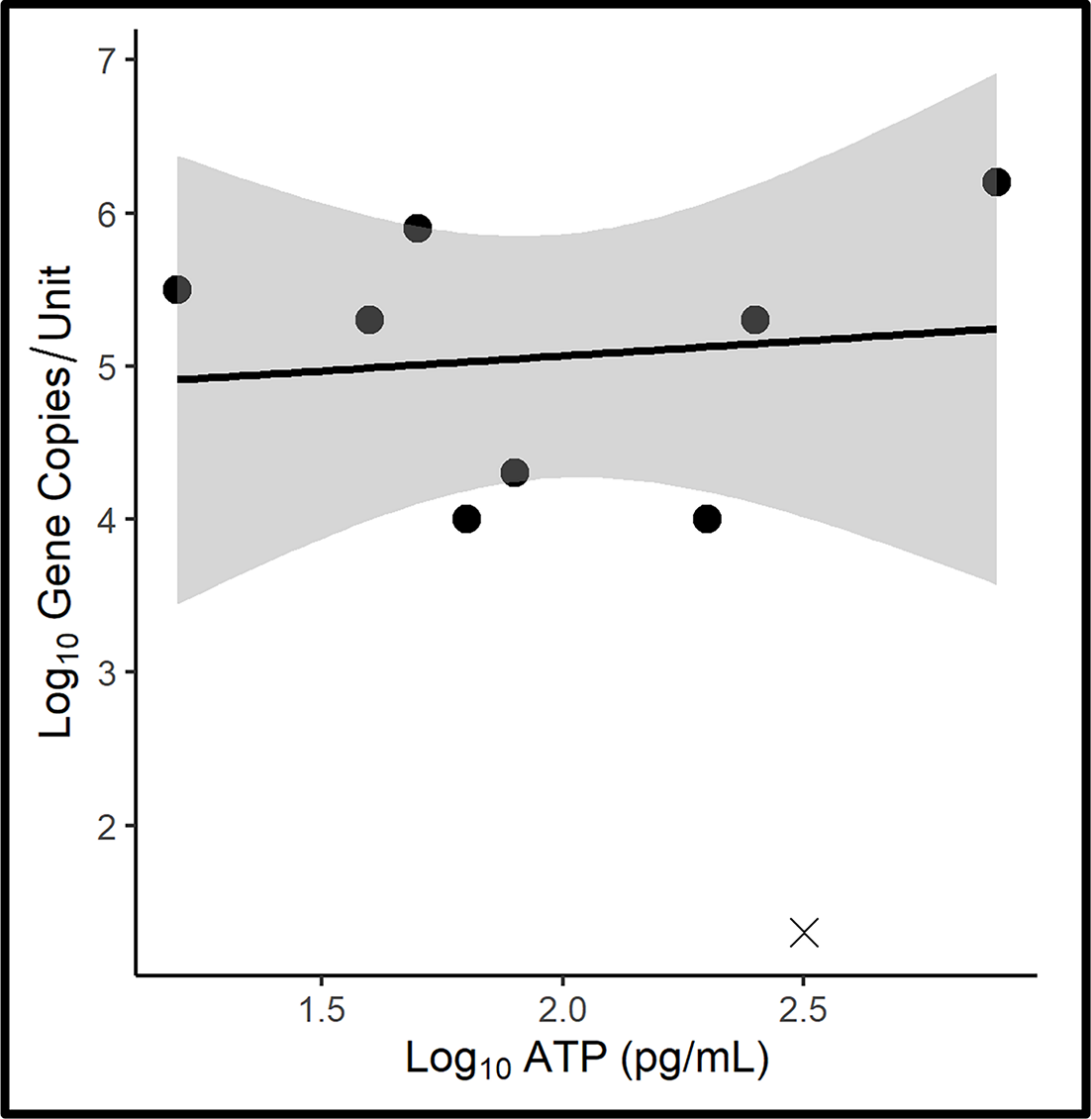
Aqueous-phase microcosm corrosion coupon surface ATP-bioburden versus qPCR-bioburden scatter plot. Grey shading along the trend line indicates the 95 % confidence interval. Datapoints denoted with ‘X’ are at or below the limit of detection (LOD) for qPCR, orATP. These values were excluded from the trendline and 95 % confidence interval as values below the LOD do not follow the same probability of repeated detection as datapoints within the range of detection.

Consistent with elevated bioburdens detected in the fuel–water interface zone, biofilm accumulation on corrosion coupons was heaviest in the same zone (Fig. 5). As seen on the scatter plot [Fig. 6, *y*=0.18*x*+6.5, *r*^2^: 0.01 (where *y* is log_10_ GC ml^−1^ and *x* is log_10_ [cATP] pg ml^−1^)], there was no significant correlation between qPCR and ATP-based bioburdens (*r*=−0.20; *r*_crit[7, α=0.05]_=0.67). The ATP-bioburden (2±0.54 log_10_ pg cm^−2^) averaged two orders of magnitude less than the qPCR-bioburden (4±2.2 log_10_ GC cm^−2^) – indicating that >90 % of the microbes in the biofilm were dormant.

**Fig. 7.**
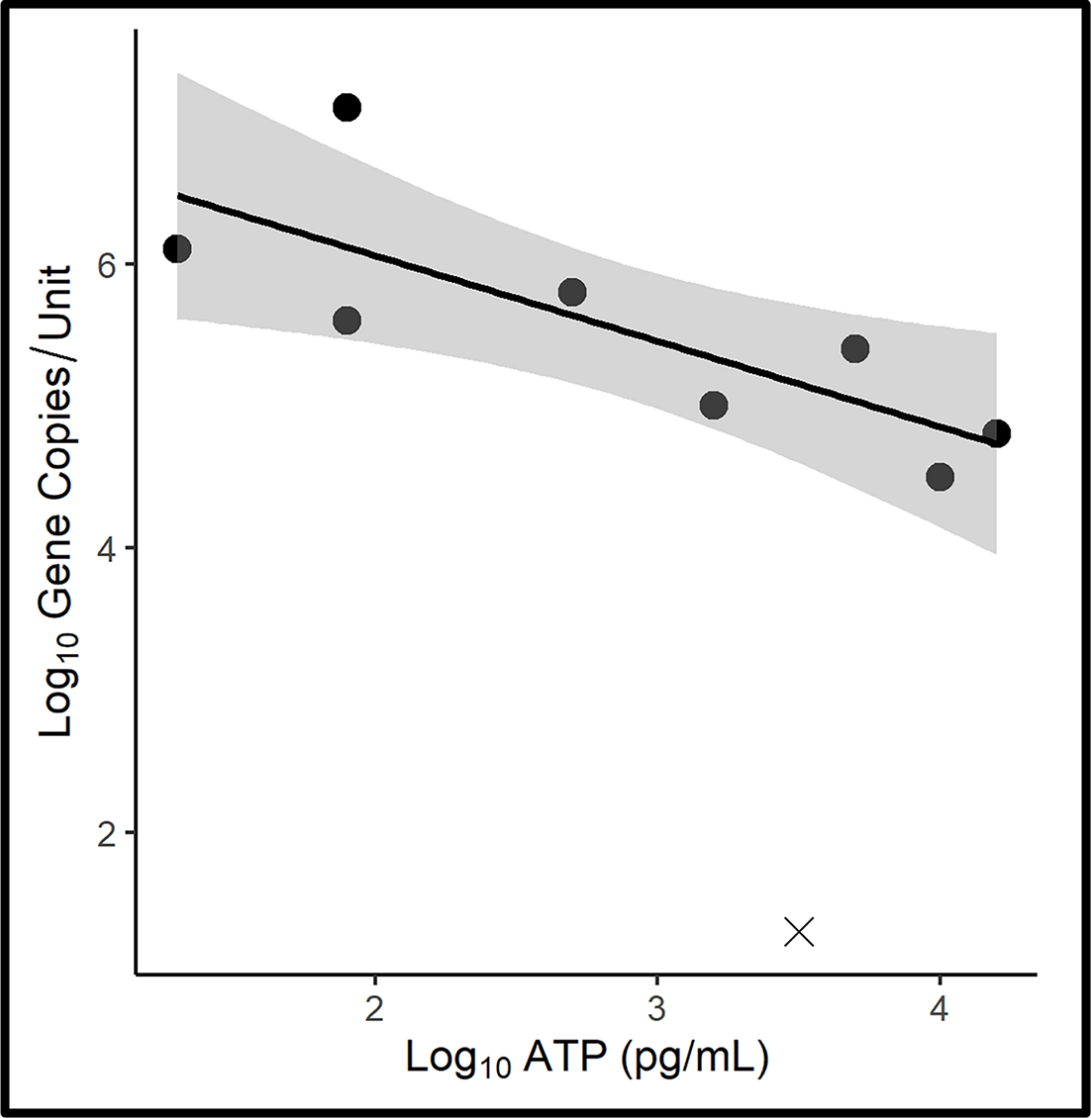
Interface microcosm corrosion coupon surface ATP-bioburden versus qPCR-bioburden scatter plot. Grey shading along the trend line indicates the 95 % confidence interval. Datapoints denoted with ‘X’ are at or below the limit of detection (LOD) for qPCR or ATP. These values were excluded from the trendline and 95 % confidence interval as values below the LOD do not follow the same probability of repeated detection as datapoints within the range of detection.

A scatter plot of the interface zone corrosion coupon surfaces is shown in Fig. 7 [regression equation: *y*=−0.6*x*+7.26, *r*^2^: 0.62; where *y* is log_10_ GC ml^−1^ and *x* is log_10_ [cATP] (pg ml^−1^)]. The qPCR-bioburdens on interface zone corrosion coupon surfaces were 5±1.6 log_10_ GC cm^−2^. As for aqueous-phase surfaces, this was two orders of magnitude greater than the ATP-bioburdens (3±1.0 pg cm^−2^). The correlation between qPCR and ATP-based bioburdens was not significant (*r*=−0.55; *r*_crit[7, α=0.05]_=0.67). The raw, corrosion coupon surface bioburden data are provided in Table S5. Again, the data suggested that a substantial proportion of the microbes within biofilms were dormant.

## Conclusions

Data from two microbiological parameters were compared to assess whether qPCR and ATP-bioburdens correlated with one another. In liquid samples containing planktonic populations, the correlation coefficients between the two parameters were significant at the 95 % confidence level. However, categorical score agreement was ≥60 % for only aqueous-phase samples. These results suggest that reliance on single microbiological parameter trends can be adequate for routine condition monitoring purposes. When diagnosing microbial contamination or assessing biodeterioration risk, data for multiple microbiological parameters can contribute to a better understanding of both bioburden and the population’s physiological state. As this was the first reported evaluation of the relationship between ATP and qPCR data for fuel and fuel-associated water, additional research is needed to better understand the factors contributing to differences in microbial bioburdens determined by ATP testing and qPCR testing, respectively. ATP testing provides a measure of metabolically active bioburden in <10 min. Because ASTM Test Method D7687 can be performed at the sample collection site, corrective actions can be initiated within minutes after sample collection.

When additional information about the composition of the contaminant population is available, the qPCR described in this paper can be used to differentiate between bacterial and fungal bioburdens. Even greater granularity can be obtained by using one or more of the primers listed in ASTM Guide D8412 (Table 1) [14] instead of the two used in this investigation. A growing literature reporting the relationships between detected microbial OTUs and observed fuel and fuel system biodeterioration can provide the basis for improved biodeterioration strategies.

## supplementary material

10.1099/acmi.0.000695.v4 Uncited Table S1.

## Abbreviations

AEC: adenylate energy charge
ANOVA: analysis of variation
ATP: adenosine triphosphate
[cATP]: cellular ATP concentration
Cn: C- carbon; n - number of carbon atoms in molecule
CV: coefficient of variance
GC: gene copies
LOD: limit of detection
OTU: operational taxonomic unit
PCR: polymerase chain reaction
qPCR: quantitative PCR
RLU: relative light units
[tATP]: total ATP concentration
TF: total fungal
TP: total prokaryote
