# Supplementary material for: The relationship between microbial population ATP and quantitative PCR bioburdens in diesel fuel microcosms
Source: Access Microbiol. 2024 Jul 4;6(7):000695.v4. doi: 10.1099/acmi.0.000695.v4 (PMC11316577; doi:10.1099/acmi.0.000695.v4)
Supplement: Uncited Table S1. [file acmi-6-00695-s001.pdf]

ACMI-D-23-00167

Supplemental Tables

The Relationship between microbial population adenosine triphosphate and quantitative polymerase chain reaction bioburdens in diesel fuel microcosms

Table S1. qPCR repeatability precision.

a. Total prokaryote (TP) qPCR repeatability standard deviation

Sample	TP (Log_{10} GC mL^{-1})				
	R1	R2	R3	AVG	s
1	4.78	4.58	4.68	4.68	0.10
2	6.32	6.44	6.37	6.38	0.06
3	4.60	4.51	4.28	4.46	0.17
4	5.18	5.54	6.15	5.63	0.49
5	4.62	4.72	4.44	4.59	0.14
$\bar{X}$	5.10	5.16	5.18	5.15	0.32
s	0.72	0.83	1.00	0.83	0.35
df	4.00	4.00	4.00		
SS	2.09	2.74	3.97		
$\bar{\bar{X}}$	5.15				

b. Total fungal (TF) qPCR repeatability standard deviation

Sample	TF (Log_{10} GC mL^{-1})				
	R1	R2	R3	AVG	s
1	4.34	4.29	4.62	4.42	0.18
2	3.07	3.01	3.18	3.09	0.09
3	3.04	3.25	3.22	3.17	0.11
4	5.37	5.41	5.42	5.40	0.03
5	4.28	4.14	4.20	4.21	0.07
6	3.52	3.74	3.70	3.65	0.12
$\bar{X}$	3.94	3.97	4.06	3.99	
s	0.90	0.86	0.87	0.88	
df	5	5	5	5	
SS	4.08	3.72	3.79		
$\bar{\bar{X}}$	3.91				

Table S2. Aqueous-phase cATP (Log_{10} pg mL^{-1}) and qPCR (Log_{10} GC mL^{-1}) test results.

Sample	[cATP]	TP	TF	TP+TF
3	3.31	5.63	1.00	5.63
4	3.34	6.09	3.08	6.09
5	3.23	3.21	3.27	3.54
10	3.19	5.15	1.00	5.15
11	-0.23	3.33	2.73	3.43
19	3.64	5.81	1.00	5.81
21	3.09	4.27	2.81	4.29
22	2.26	4.87	1.00	4.87
24	3.05	4.05	3.78	4.24
28	2.99	4.27	1.00	4.27
30	4.18	6.02	3.52	6.02
35	3.05	1.00	1.00	1.30
36	0.84	1.00	1.00	1.30
37	1.69	4.51	1.00	4.51
39	3.62	5.29	3.43	5.29
40	4.32	5.68	4.55	5.72
41	1.37	1.00	1.00	1.30
44	3.47	6.40	1.00	6.40
45	2.44	3.33	1.00	3.33
46	2.83	4.43	2.52	4.44
47	2.20	3.09	1.00	3.09
49	2.62	4.63	2.63	4.64
54	3.33	4.43	4.23	4.64
55	3.52	1.00	5.16	5.16
56	3.46	5.70	2.43	5.70
57	3.63	5.60	1.00	5.60
58	3.26	4.21	1.00	4.21
64	3.26	1.00	1.00	1.30
66	3.80	5.66	2.91	5.66
67	3.53	4.91	2.81	4.92
72	3.04	4.08	1.00	4.08
74	1.76	4.09	1.00	4.09
75	3.44	1.00	1.00	1.30
76	2.95	2.94	2.73	3.15
77	2.19	3.80	1.00	3.80
78	1.00	1.00	3.04	3.05
80	3.39	4.28	3.62	4.36
81	3.54	3.74	2.60	3.77
91	0.97	1.00	1.00	1.30

Table S2 Continued

Sample	[cATP]	TP	TF	TP+TF
92	2.08	1.00	1.00	1.30
93	3.24	3.62	2.56	3.66
94	3.13	4.31	1.00	4.31
96	3.01	4.91	1.00	4.91
98	2.17	1.00	1.00	1.30
100	1.85	4.04	1.00	4.04
101	1.59	1.00	1.00	1.30
102	3.56	4.12	1.00	4.12
103	2.64	1.00	1.00	1.30
104	3.06	1.00	1.00	1.30
105	2.86	4.54	1.00	4.54
107	2.96	5.21	1.00	5.21
110	3.81	1.00	4.86	4.86
111	1.68	1.00	1.00	1.30
112	4.05	4.35	2.94	4.37
113	3.82	3.14	1.00	3.14
114	2.00	1.00	1.00	1.30
115	2.27	1.00	1.00	1.30
116	3.53	7.19	5.08	7.19
122	2.85	5.48	3.42	5.49
123	3.76	4.30	6.27	6.27
124	3.35	6.85	5.60	6.87
125	2.61	3.60	2.97	3.69
126	2.24	1.00	2.67	2.68
127	2.94	4.16	2.46	4.17
128	3.99	5.44	3.26	5.44

Table S3. Fuel-phase cATP (Log_{10} pg mL^{-1}) and qPCR (Log $_{10}$ GC mL^{-1}) test results.

Log ATP (pg/unit)	Log TP+TF (GC/unit)	ATP	qPCR	ATP = qPCR	ATP > qPCR	qPCR > ATP
2.03	2.78	3	3	1	0	0
2.49	2.33	3	3	1	0	0
2.47	4.86	3	5	0	0	1
2.44	4.12	3	5	0	0	1
2.00	1.30	3	1	0	1	0
2.94	3.51	3	5	0	0	1
3.08	4.21	5	5	1	0	0
2.45	4.04	3	5	0	0	1
0.71	1.30	1	1	1	0	0
**Totals**				4	1	4
**Percentages**				44%	11%	44%

Table S4. Fuel-water interface cATP (Log_{10} pg mL^{-1}) and qPCR (Log $_{10}$ GC mL^{-1}) test results.

Log [cATP] (pg mL^{-1})	Log TP+TF (GC mL^{-1})	ATP	qPCR	ATP = qPCR	ATP > qPCR	qPCR > ATP
4.77	8.72	3	5	0	0	1
4.87	8.07	3	5	0	0	1
4.80	6.40	3	5	0	0	1
4.59	7.70	3	5	0	0	1
2.20	1.30	1	1	1	0	0
3.99	7.93	3	5	0	0	1
3.94	9.44	3	5	0	0	1
3.85	5.26	3	5	0	0	1
4.30	7.13	3	5	0	0	1
3.56	7.11	3	5	0	0	1
4.13	1.30	3	1	0	1	0
3.11	7.17	3	5	0	0	1
4.05	8.25	3	5	0	0	1
3.88	7.52	3	5	0	0	1
5.54	7.91	5	5	1	0	0
2.19	1.30	1	1	1	0	0
3.34	1.30	3	1	0	1	0
5.27	5.40	5	5	1	0	0
2.93	7.80	1	5	0	0	1
3.39	4.29	3	3	1	0	0
3.83	1.30	3	1	0	1	0
**Totals**				5	3	13
**Percentages**				24%	14%	62%

Table S5. Corrosion coupon surface cATP (Log_{10} pg cm^{-2}) and qPCR (Log_{10} GC cn^{-2}) test results.

a. **Aqueous-phase surfaces**

Log [tATP] (pg cm^{-2})	Log TP+TF (GCcm^{-2})	ATP	qPCR	ATP = qPCR	ATP > qPCR	qPCR > ATP
2.91	6.23	1	5	0	0	1
1.17	5.51	1	5	0	0	1
1.88	4.34	1	3	0	0	1
1.71	5.92	1	5	0	0	1
1.58	5.26	1	5	0	0	1
2.49	1.30	1	1	1	0	0
2.25	3.99	1	3	0	0	1
1.82	4.04	1	3	0	0	1
2.44	5.31	1	5	0	0	1
**Totals**				1	0	8
**Percentages**				11%	0%	89%

b. **Interface zone surfaces**

Log [tATP] (pg cm^{-2})	Log TP+TF (GCcm^{-2})	ATP	qPCR	ATP = qPCR	ATP > qPCR	qPCR > ATP
3.67	5.36	3	5	0	0	1
1.92	5.57	1	5	0	0	1
4.24	4.77	3	3	1	0	0
2.72	5.85	1	5	0	0	1
1.89	7.14	1	5	0	0	1
4.05	4.51	3	3	1	0	0
1.26	6.10	1	5	0	0	1
3.50	1.30	3	1	0	1	0
3.17	4.96	3	3	1	0	0
**Totals**				3	1	5
**Percentages**				33%	11%	56%
